# Implementing guidelines for follow-up after surgery with ventilation tube in the tympanic membrane in Norway: a retrospective study

**DOI:** 10.1186/1472-6815-13-2

**Published:** 2013-01-08

**Authors:** Bjarne Austad, Irene Hetlevik, Vegard Bugten, Siri Wennberg, Anita Helene Olsen, Anne-Sofie Helvik

**Affiliations:** 1 General Practice Research Unit, Department of Public Health and General Practice, Norwegian University of Science and Technology (NTNU), Trondheim, Norway and Sjøsiden Medical Centre, 7491, Trondheim, Norway; 2 General Practice Research Unit, Department of Public Health and General Practice, NTNU, Trondheim, Norway; 3 Department of Neuromedicine, Faculty of Medicine, NTNU, Trondheim, Norway and Department of Ear, Nose and Throat, Head and Neck Surgery, St. Olavs Hospital, Trondheim, Norway; 4 Audiological Department, Department of Ear, Nose and Throat, Head and Neck Surgery, St. Olavs Hospital, Trondheim, Norway; 5 General Practice Research Unit, Department of Public Health and General Practice, NTNU, Trondheim, Norway and Department of Ear, Nose and Throat, Head and Neck Surgery, St. Olavs Hospital, Trondheim, Norway

**Keywords:** Implementation, Coordination, Clinical guidelines, Primary care, General practice, Ventilation tube, Otitis media

## Abstract

**Background:**

When clinical guidelines are being changed a strategy is required for implementation. St. Olavs University Hospital in Norway modified their guidelines for the follow-up care of children after insertion of ventilation tubes (VT) in the tympanic membrane, transferring the controls of the healthiest children to General Practitioners (GPs). This study evaluates the implementation process in the hospital and in general practice by exploring two issues: 1) Whether the hospital discharged the patients they were supposed to and 2) whether the children consulted a GP for follow-up care.

**Methods:**

A retrospective observational study was performed at St. Olavs University Hospital, Norway and general practice in Mid-Norway. Children under the age of 18 who underwent insertion of VT between Nov 1st 2007 and Dec 31st 2008 (n = 136) were included. Degree of guideline adherence at the hospital and in general practice was measured.

**Results:**

The hospital adhered to the guidelines in two-thirds (68.5%) of the patients, planning more patients for follow-up by their GP than recommended in the guidelines (25.8% vs. 12.4%). All except one contacted their GP for control. In total 60% were referred back to specialist health services within two years.

**Conclusions:**

The methods for guideline implementation were successful in securing consultations for follow-up care in general practice. Lack of guideline adherence in the hospital can partly be explained by the lack of quality of the guideline. Further studies are needed to evaluate the quality of controls done by the GPs in order to consider implications for follow-up after VT surgery.

## Background

General practitioners (GPs) receive numerous clinical guidelines from hospitals and others, developed with good intentions for quality improvement. Some guidelines will not be implemented and will therefore not have the desired effect [1-3]. In Norway GPs have the role as gate-keepers, expected to refer to secondary care only what cannot be handled in primary care. A Coordination Reform between the hospitals and primary care was set into practice in 2012 [4]. One of the aims has been to transfer obligations and responsibility from secondary to primary care. Development and implementation of clinical guidelines are regarded to be among the major strategies for knowledge transfer [5]. This makes it utterly important to understand how the implementation process works and identify barriers against implementation [6-9].

Children with otitis media with effusion or recurrent otitis media are frequently treated with a ventilation tube (VT) placed in the tympanic membrane [10-12]. Little research has been done on the follow-up care after this kind of surgery. In 2008 the Swedish Council on Health Technology Assessment (SBU) completed a systematic literature review focusing on the documentation of VT treatment. They could not conclude how and when children with inserted VTs best should be followed up [13]. In Norway follow-ups of VTs are mostly done by Ear-Nose-Throat (ENT) specialists [14], i.e. on a more expensive health care level than general practice.

St. Olavs University Hospital in Mid-Norway has modified their guidelines for follow-up after VT surgery recommending that children with normal or minor hearing loss should get follow-ups from their GP, first at six months and again at 18 months after surgery. The GP’s received a simple guideline on how to handle complications, such as a plugged tube with ear drops for two weeks followed by another control by the GP, and also to refer back to a specialist if the VT was not rejected within 18 months. Children with medical syndromes, hearing loss above 30 dB in at least one frequency (0.5-1-2-4 kHz) in the worst ear, or unresolved hearing (not audiological tested, but with suspected hearing loss), were still recommended to have their follow-ups at the outpatient clinic [15]. Point of time for control at the outpatient clinic could vary depending on the severity of the disease. Arguments for revision of guidelines were cost-effectiveness and to save outpatient clinic resources. However, one worried that children discharged from the hospital might forget controls due to lack of summoning in general practice.

Lack of adherence to guidelines is well known, both in relation to process [2,16] and outcome [17]. Efforts have been made to explore the phenomenon without conclusion [3,5,18,19]. Implementation research has revealed that multifaceted methods for guideline implementation are more successful than use of single methods [20,21]. As a consequence, multifaceted strategies were used for implementation in this study, both at the hospital and in general practice.

To implement the guidelines at the hospital they were: (1) developed by physicians at the ENT department in order to establish ownership, (2) made accessible in the hospital’s internal quality system, and (3) repeated several times during daily work at the Department. To implement the guidelines in primary care: (1) the Head of the ENT Department verbally informed the GPs in a mandatory medical meeting for GPs. After discussing the guidelines the GPs agreed to do the follow-ups as recommended in the guidelines; (2) the GPs received written procedures on how the controls should be performed and how to handle complications [15]; and (3) parents were informed verbally and in writing about the new procedure and instructed to make the appointments with their GP themselves [22].

This study explores the process of implementation of the clinical guideline for follow-up after VT surgery. We focus on whether the hospital discharged the patients they were supposed to according to the guidelines and whether the children consulted their GP for follow-up. Audiological outcome or complications are not focused and, thus, have not been assessed.

## Methods

Inclusion criteria were insertion of a VT in the tympanic membrane in minimum one ear in patients under the age of 18 at St. Olavs University Hospital the first 14 months after the change of guidelines; i.e. between Nov 1st 2007 and Dec 31^st^ 2008. A total of 137 children underwent surgery in this period and 136 children were relevant for the study. One was excluded because of a co-existing severe disease.

The implementation strategy both in the hospital and in general practice took place in 2007. The parents received the verbal and written information at time of surgery. Nearly two years after surgery (24 ± 3 months) all 136 children with parents/guardians were invited by letter to participate in this evaluation study exploring adherence to clinical guidelines. The invitation included a self-report questionnaire and an appointment for an audiological consultation. The parents and children completed the self-report questionnaire latest at the time of consultation.

The participants were included after informed written consent. Due to Norwegian regulations parents/guardians had to give consent on their own behalf and on behalf of children under the age of 16. Children and adolescents 16 years and older consented on behalf of themselves. The study was approved by the Regional Ethics Committee in Sør-Trøndelag (2009/155-2) and the Norwegian Social Science Data Service (NSD).

Information about the audiological test prior to surgery was obtained from the medical record when the patients were included in the study. The pure tone thresholds at 0.5-1-2-4 kHz form the mean threshold [23]. To be included in the analysis of the mean threshold at least three of these frequencies had to be present. If a preoperative audiological test was lacking, the patient record was read carefully with the purpose of identifying suspected hearing loss or unresolved hearing.

The questionnaire used in the study included 16 questions, among them the number of VT surgeries they had gone through, the date of their most recent surgery, location and frequency of follow-ups after surgery and potential referral back to the hospital. Furthermore, they were asked to provide socio-demographic data, including parental education and occupation. The questions had been pilot tested among employees at the ENT department before used in the study.

### Statistical methods

Data was read optically, quality assured and then analyzed with SPSS 19. Categorical data were assessed with chi-square test, while normally distributed continuous data were assessed with *t*-test and ANOVA. The groups of children scheduled for follow-up by the outpatient clinic (n = 60) and by private ENT clinics (n = 6) were analyzed as one group, the specialist health service group. Most of the children with medical syndromes also had hearing loss or unresolved hearing; they have been categorized only into a medical syndromes subgroup.

## Results

A total of 89 children (65.4%) completed the audiological consultation. Two did not deliver the questionnaire. Data characteristics are listed in Table 1. There were no statistical significant differences between gender and age of the participants, mean threshold in the worst ear prior to surgery or parents’ education in the GP group compared to the specialist health service group.

**Table 1:** Participants sex, age, time after surgery, audiological status, and parents’ level of education

	
**Female** (%)	41.6%
**Male** (%)	58.4%
**Age at examination** Mean (min-max)	6.1 years (3.0 – 16.4)
**Time after surgery** Mean (min-max)	2.1 years (1.8 – 3.1)
**Ventilation tube surgery more than once** n (%)	50 (56.2%)
**Audiological tests before surgery** n (%)	
Pure tone, speech or play audiometry	45 (50.6%)
Informal hearing tests	6 (6.7%)
Not hearing tested	38 (42.7%)
**Age hearing tested** Mean (min-max)	4.9 years (1.6 – 12.7)
**Age not hearing tested**	2.8 years (0.8 – 14.4)
**Mean threshold (0.5-2 kHz) before surgery worst ear** Mean (min-max)	31.8 dBHL (10 – 83.8)
**Education above high-school level mother** n (%)	65 (73%)
**Education above high-school level father** n (%)	55 (61.8%)

dBHL, decibel hearing level; kHz, kiloHertz.

Table 2 gives information about the discrepancy between where follow-ups should have taken place according to the clinical guidelines and where follow-ups were planned to take place when the children left the hospital. The hospital adhered to the guideline in 61 (68.5%) of the children. Despite the new guidelines, eight participants were scheduled for follow-ups with the specialist health services instead of the GPs. Of those eight, four had been referred by local hospitals or private ENT clinics, and returned to those hospitals and clinics for their follow-up appointments, one had VT surgery more than four times and the last three had minor or no extra complications.

**Table 2:** Hospital plan versus guideline recommendation for follow-up of patients after surgery

		**Hospital plan for follow-up**	
		**General practitioner (n = 23)**	**Specialist health service (n = 66)**
**Hospital guidelines for follow-up**	**General practitioner** (n = 11)	3	8
	**Specialist health service** (n = 78)	20	58

Table 3 explores the hospital’s plan for follow-up of the 78 children recommended for specialist health service follow-up in the guidelines. In these cases, the hospital did not adhere to the guidelines for 20 (25.6%) children.

**Table 3:** Hospital plan for follow-up of the subgroups of patients that according to the guidelines should be followed-up by specialist health service (n = 78)

		**Hospital plan for follow-up**	
		**General Practitioner**	**Specialist health service**
**Subgroups of patients which in accordance to the guidelines should be followed by the specialist health service**	**Medical Syndrome** n (% of group)	0 (0%)	16 (100%)
	**Hearing loss ≥ 30 dB** n (% of group)	11 (32.4%)	23 (67.6%)
	**Unresolved or suspected hearing loss** n (% of group)	9 (32.1%)	19 (67.9%)

Table 4 reports where the patients according to the questionnaire actually went for follow-up. A total of 41 (10 + 31) (47.7%) consulted their GP for VT control, and of those 25 (61.0%) were referred back to the specialist health service. Among the 20 (7 + 13) children scheduled for follow-ups with and actually had the VT controlled by a GP, 12 (60%) were referred back to the specialist health service. Data concerning reasons for being seen by a specialist, even when assigned to the GP for follow-up, could not be obtained.

**Table 4:** The accomplished follow-up of the patient after surgery compared to hospital plan for follow-up

		**Hospital plan for follow-up**		
		**General Practitioner**	**Specialist health service**	**Total**
		**(n = 23)**	**(n = 63)**^**1**^	**(n = 86)**
**The accomplished follow-up**	No follow-up	1	5	6
	Only General Practitioner	7	3	10
	Only specialist health service	2	37	39
	Both General Practitioner and specialist health service	13	18	31

^1^Missing information from 3 respondents, all in the specialist health service group.

Six children did not obtain control of the VT at all, one (4.3%) in the GP group and five (7.6%) in the specialist health service group. The one not controlled in the GP group was explained by lack of information about control being necessary; none answered that they forgot to contact the GP for control themselves. In the specialist health service group reasons for not controlling the VT were: lack of information (one), felt no need for control (one) and other reasons (three). Other reasons were specified as: patient ill (one), doctor ill (one) and not summoned (one).

## Discussion

The hospital adhered to the guidelines for two-thirds of the children, but to all children who had medical syndromes. According to the guidelines only 11 of 89 children were eligible for follow-ups by the GP, but the hospital planned 23 for GP follow-ups. Of these, all except one consulted their GP after VT surgery.

Strength in this study is the inclusion of all children who underwent VT-surgery, not only those planned to get follow-up care from their GP. However, the response rate to this study - 65.4% - was somewhat low, and many of the children did not have an audiological evaluation before surgery. The best method for research on guideline implementation is a randomized controlled design with the purpose of giving valid information about the chosen method’s efficacy. The evaluation done in this study is however more compatible with how things actually take place in clinical practice and in the collaboration between the different levels in the health care system; i.e. the study contributes with information about effectiveness related to guideline implementation.

A broad variety of guideline implementation strategies have been described [6,8,24-26]. However, as “none of the approaches is superior for all changes in all situations; we probably need them all” according to Grol and Grimshaw [9]. Multifaceted methods, motivation of physicians, repetition of recommendations and guideline availability at consultation are demonstrated to be effective [1,5,20,27,28]. In our study, facilitators for implementation in the hospital were the physicians’ ownership to the guidelines and repetition of the recommendations. The guidelines were partly initiated because of increasing waiting lists at the outpatient clinic. Other studies have shown that administrative motivated guidelines can be difficult to implement into practice [9].

### Implementation at the hospital

The results may give the impression that guideline implementation at the hospital did not succeed. However, all children with medical syndromes did get follow-ups according to the guidelines, so the divergence concerns those with impaired or unresolved hearing. Many of the children were so young at time of surgery that audiological evaluation was not possible; thereby leaving a large amount with unresolved hearing. Hearing loss > 30 dB in at least one frequency appears quite frequently amongst those in need of VT surgery [29,30]. Therefore, it is possible that after a clinical assessment the surgeon regarded the guidelines as partly being inadequate for allocating follow-ups. For instance the guideline does not mention how to define “unresolved hearing” and how to handle children who have been referred from local hospitals or private ENT clinics, leaving these patients to the surgeons’ judgment.

The main point in the new guidelines was to delegate controls to the GPs. This was clearly implemented as the surgeons did not end too few as feared in advance, but too many according to the guidelines. Of the eight children that despite the guideline recommendations were planned for follow-up by the specialist health service, it looks as if the surgeons had valid reasons for this decision in most cases. Therefore, two-thirds concordance may be as successful as could be expected with guidelines not being sufficiently detailed to guide practice in all cases.

### Implementation in general practice

The fear that parents should forget to take their children to consult the GP for VT control seems groundless in our material. In Norway, a list-based system in primary care was established in 2001 so the participants knew which GP to consult. This fact, in combination with leaving the responsibility to parents for making the appointments themselves may be reasons for the successful implementation of this routine. Other reasons may be that complications, ear-infections and questions concerning the VT work as “reminders” to control the VT [31].

Even if data concerning reasons for referral back to the specialist health service could not be obtained, it is reasonable to expect that persisting tubes, recurrence of the disease, or complications could be among the major explanations. However, some GPs might have experienced uncertainty in controls of the VT, and this could have influenced the high referral rate back to the specialist health service. The implementation strategy included one meeting, and the written procedure on how to control the VT was sent only once to the GPs. Lack of repetition may represent a barrier towards implementation [5] and contribute to the referral rate back to the hospital; after all most GPs do not control many children with VT. One suggestion could be to include the guideline in the discharge report from the hospital in order to make the GP feel more secure in relation to the procedures.

### Shared care

One-third of the children planned for specialist health service follow-up also went to their GP to control the VT. We do not know the reasons, but some possibilities may be ear infections, late summoning from the specialist health service or questions after surgery in combination with easier availability at the GP than at the hospital. In addition, it might be that one control took place at the hospital as planned and the patient thereafter was recommended to have follow-ups by GPs. This finding may also indicate that construction of strictly separate recommendations for follow- ups may not be realistic, some degree of shared care will occur, and may also be wanted for different reasons.

Our material is from a university hospital where the sickest children in need of VT in the region are treated. If the study was committed on a local hospital or a private ENT clinic, the percentage of patients who could be controlled by the GP would presumably be higher.

## Conclusion

We have examined the process of implementation of new guidelines for follow-up after surgery with VT in the tympanic membrane. Audiological outcome or complications have not been assessed. The hospital adhered to the guidelines in two-thirds of the patients. Lack of guideline adherence can partly be explained by the lack of quality of the guideline. The main point of the guideline was to have more controls in primary care. This was implemented as the hospital discharged more patients than the guidelines suggested.

The implementation was also successful when it comes to patients consulting their GP for controls. Further research is needed to assess the quality of GPs controls.

## Competing interest

The authors declare that they have no competing interests.

## Authors' contributions

BA participated in design of the study, analysis and interpretation of the data and drafted the manuscript. IH, VB and ASH participated in design of the study, supervising and editing the manuscript. AHO and SW contributed in data collection. All authors read and approved the final manuscript.

## Authors' information

BA is a general practitioner and research fellow. IH is professor in general practice and former general practitioner. VB is an Ear-Nose-Throat surgeon and associate professor. AHO and SW are audiologists. ASH is RN, Dr. Philos and researcher.

## Pre-publication history

The pre-publication history for this paper can be accessed here:

http://www.biomedcentral.com/1472-6815/13/2/prepub
